# Visceral and Dysfunctional Adiposity Indices as Predictors of Insulin Resistance and Metabolic Syndrome in Women with Polycystic Ovary Syndrome: A Cross-Sectional Study

**DOI:** 10.3390/medicina61030424

**Published:** 2025-02-28

**Authors:** Betül Keyif, Ali Yavuzcan

**Affiliations:** 1 Department of Obstetrics and Gynecology, Faculty of Medicine, Düzce University, 81620 Düzce, Turkey; 2 Department of Obstetrics and Gynecology, Faculty of Medicine, Sağlık Bilimleri University, Ankara Bilkent City Hospital, 06800 Ankara, Turkey; draliyavuzcan@yahoo.com

**Keywords:** adiposity indices, metabolic risk assessment, insulin resistance, metabolic syndrome

## Abstract

*Background and Objectives*: Polycystic ovary syndrome (PCOS) is one of the most common endocrine disorders in women of reproductive age. Women with PCOS often have metabolic disorders such as insulin resistance (IR), type 2 diabetes (T2DM), obesity, and metabolic syndrome (MetS). The assessment of visceral adiposity and dysfunctional adipose tissue is crucial for understanding the metabolic risks associated with PCOS. The visceral adiposity index (VAI) and the dysfunctional adiposity index (DAI) are two novel metabolic indices that more specifically assess adipose tissue dysfunction and visceral fat accumulation. This study aimed to evaluate the clinical utility of VAI and DAI as predictive markers for metabolic complications such as insulin resistance (IR) and metabolic syndrome (MetS) in women with PCOS. *Materials and Methods*: This cross-sectional study included 92 women diagnosed with PCOS based on the 2023 revised Rotterdam criteria, as well as 68 healthy controls. Anthropometric and biochemical parameters, including fasting glucose, insulin, lipid profile, and hormonal markers, were recorded. VAI and DAI were calculated using established formulas derived from previous validated studies. *Results*: The mean VAI in PCOS patients was 4.26 ± 3.23, compared to 2.61 ± 1.92 in controls (*p* = 0.003). The mean DAI in PCOS patients was 3.00 ± 1.86, while in controls it was 1.86 ± 1.22 (*p* = 0.003). Both VAI (Area Under the Curve [AUC] = 0.639) and DAI (AUC = 0.635) did not demonstrate statistically significant diagnostic performance for PCOS itself, but they were strongly associated with metabolic disturbances within the PCOS group. VAI and DAI values were significantly elevated in PCOS patients with IR (*p* < 0.001) and MetS (*p* < 0.001). For MetS in PCOS patients, VAI demonstrated the highest predictive ability, with an AUC of 0.87 and a cutoff of 4.73 (sensitivity 62%, specificity 92%), while DAI had an AUC of 0.86 with a cutoff of 2.44 (sensitivity 74%, specificity 80%). Regarding IR in PCOS patients, VAI had an AUC of 0.75 with a cutoff of 2.56 (sensitivity 82%, specificity 56%), while DAI had an AUC of 0.74 with a cutoff of 1.59, showing a sensitivity of 82% and a specificity of 55%. *Conclusions*: Although VAI and DAI are not suitable for diagnosing PCOS, they provide valuable insights into the metabolic risks associated with the condition. VAI and DAI can serve as promising biomarkers for identifying IR and MetS risk in women with PCOS. Their integration into clinical practice may facilitate the early detection of cardiometabolic complications, offering a more specific metabolic risk assessment compared to traditional anthropometric measures.

## 1. Introduction

Polycystic ovary syndrome (PCOS) is one of the most common endocrine disorders in women of reproductive age, affecting approximately 6–15% of women worldwide depending on diagnostic criteria [1,2]. This condition not only significantly impacts reproductive health but also increases the risk of metabolic complications. Women with PCOS frequently exhibit metabolic disorders such as insulin resistance (IR), type 2 diabetes mellitus (T2DM), obesity, and metabolic syndrome (MetS). These metabolic disturbances contribute to an increased risk of cardiovascular disease (CVD), independent of body mass index (BMI). The association between PCOS and increased CVD risk is primarily attributed to abdominal fat accumulation and visceral adipose tissue dysfunction [3,4].

Traditionally, anthropometric measurements such as BMI, waist-to-hip ratio (WHR), and waist circumference (WC) have been used as surrogate markers for metabolic risk in PCOS. However, these measurements do not differentiate between subcutaneous and visceral fat, nor do they capture functional impairments in adipose tissue metabolism [5]. Visceral adipose tissue dysfunction plays a central role in the development of IR, chronic low-grade inflammation, and cardiometabolic complications in PCOS patients. This limitation has led to the development of alternative metabolic indices that can more accurately reflect visceral adiposity and adipose tissue dysfunction [6,7].

The visceral adiposity index (VAI) and the dysfunctional adiposity index (DAI) are two recently proposed metabolic indices aimed at providing a more refined assessment of visceral fat accumulation and adipocyte dysfunction [8]. Unlike BMI or WHR, these indices incorporate biochemical parameters, offering a more functional evaluation of metabolic health. The VAI is an established marker of abdominal obesity and IR, integrating WC, triglyceride (TG) levels, and high-density lipoprotein cholesterol (HDL-C) to predict metabolic risk. Similarly, the DAI has been developed as a more specific measure of adipose tissue dysfunction by combining WC, BMI, TG levels, and HDL-C [9,10,11].

Emerging evidence suggests that VAI and DAI may provide better discrimination of metabolic risk in PCOS than traditional anthropometric measurements alone. However, their clinical utility in PCOS remains incompletely understood, particularly in relation to IR and MetS. Therefore, this study aimed to evaluate the predictive value of VAI and DAI for identifying IR and MetS in women with PCOS and to explore their potential role as practical, accessible, and objective biomarkers in clinical practice.

## 2. Materials and Methods

### 2.1. Study Design and Ethical Approval

This retrospective cross-sectional study was conducted on patients admitted to the outpatient clinic at the Department of Obstetrics and Gynecology, Düzce University Faculty of Medicine, between October 2018 and June 2024. Ethical approval was obtained from the Düzce University Clinical Research Ethics Committee (Approval No: 2024/173). This study was performed in accordance with the principles of the Declaration of Helsinki. Written informed consent was obtained from all participants before their inclusion in this study.

### 2.2. Study Population

**Figure 1:**
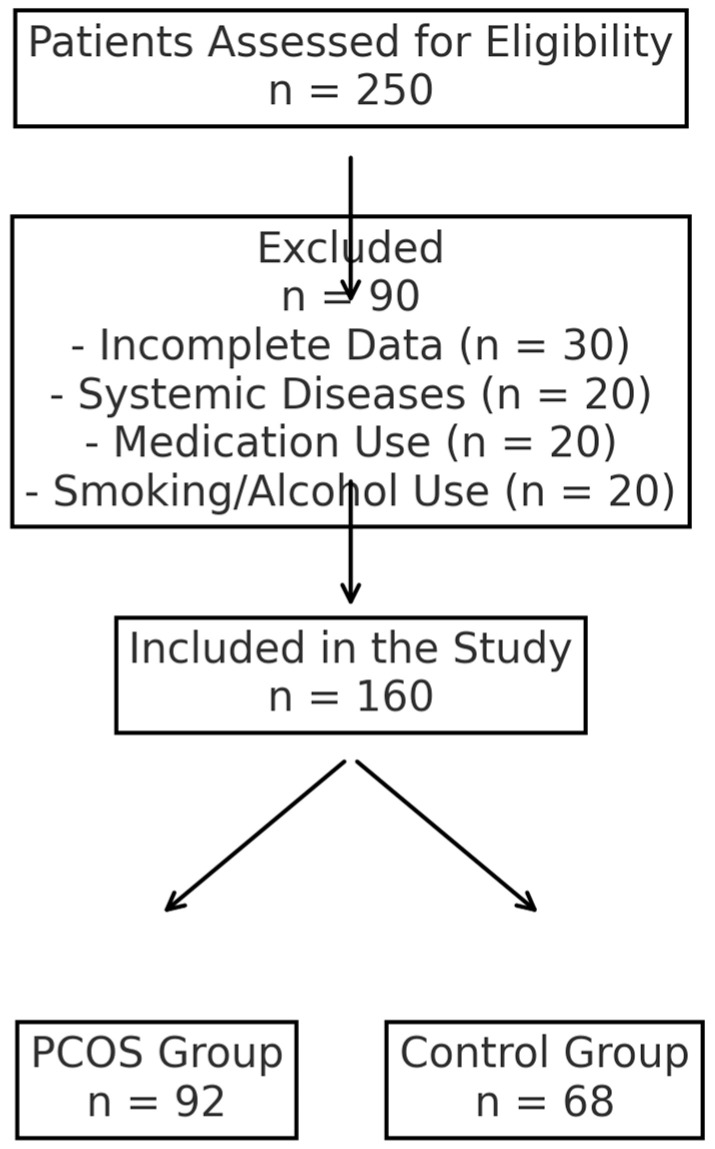
Study flowchart showing inclusion and exclusion criteria.

This study included 160 women aged 18 to 45 years, consisting of 92 patients diagnosed with PCOS based on the 2023 Rotterdam criteria and 68 age-matched healthy controls. A PCOS diagnosis was established when at least two of the following criteria were met: irregular menstrual cycles, biochemical or clinical hyperandrogenism, and ultrasound evidence of polycystic ovarian morphology [12]. The exclusion criteria included Cushing’s disease, tuberculosis, androgen-secreting ovarian tumors, adrenal hyperplasia, malignancy, decreased ovarian reserve, or systemic conditions such as thyroid dysfunction, diabetes, hepatic dysfunction, renal dysfunction, or hypertension. Patients using glucocorticoids, hormonal therapy, antidiabetic drugs, or antihyperlipidemic treatment were not included in this study. Additionally, smokers, alcohol users, and patients with incomplete medical records were excluded. The study flowchart is presented in Figure 1, illustrating the selection process of the study participants, including the inclusion and exclusion criteria applied.

### 2.3. Data Collection and Biochemical Analysis

Data were collected retrospectively from participants’ medical records. Demographic characteristics and biochemical/hormonal parameters were recorded. Venous blood samples were collected after an 8 h overnight fasting period on the second or third day of the menstrual cycle. The metabolic parameters analyzed included fasting plasma glucose (FPG), fasting insulin (FINS), total cholesterol, triglycerides (TGs), low-density lipoprotein cholesterol (LDL-C), and high-density lipoprotein cholesterol (HDL-C). Hormonal assessments included follicle-stimulating hormone (FSH), luteinizing hormone (LH), total testosterone, and thyroid-stimulating hormone (TSH) [13].

### 2.4. Calculation of Adiposity Indices and Insulin Resistance

#### 2.4.1. Visceral Adiposity Index (VAI)

VAI = [WC (cm)/(36.58 + (1.89 × BMI))] × (TG (mmol/L)/0.81) × (1.52/HDL-C (mmol/L))

The visceral adiposity index (VAI) is a gender-specific mathematical model that evaluates visceral adiposity and its role in metabolic risk. It incorporates waist circumference (WC), body mass index (BMI), triglyceride (TG) levels, and high-density lipoprotein cholesterol (HDL-C). A higher VAI score indicates increased metabolic risk and insulin resistance [14].

#### 2.4.2. Dysfunctional Adiposity Index (DAI)

DAI = [WC (cm)/(24.02 + (2.37 × BMI))] × (TG (mmol/L)/1.32) × (1.43/HDL-C (mmol/L))

The dysfunctional adiposity index (DAI) is another adiposity-based metric used to assess morphofunctional changes in adipose tissue. Similar to VAI, it incorporates WC, BMI, TG, and HDL-C, but is structured differently to provide an additional perspective on metabolic dysfunction [15].

#### 2.4.3. Homeostatic Model Assessment of Insulin Resistance (HOMA-IR)

HOMA-IR = (FPG (mmol/L) × FINS (mIU/L))/22.5

The Homeostatic Model Assessment of Insulin Resistance (HOMA-IR) is a widely used method for estimating insulin resistance. It is calculated using fasting plasma glucose (FPG) and fasting insulin (FINS) levels. Higher HOMA-IR values indicate greater insulin resistance, which is a key feature of metabolic syndrome and type 2 diabetes [16].

### 2.5. Definition of Metabolic Syndrome (MetS)

Metabolic syndrome (MetS) was defined according to the International Diabetes Federation (IDF) criteria, which require the presence of central obesity along with at least two of the following criteria: fasting plasma glucose levels greater than 100 mg/dL or a prior diagnosis of type 2 diabetes, triglyceride levels greater than 150 mg/dL or specific lipid-lowering treatment, and reduced HDL-C levels (<50 mg/dL in women) or specific therapeutic intervention for dyslipidemia [17].

### 2.6. Statistical Analysis

All statistical analyses were conducted using IBM SPSS Statistics for Windows (version 29, SPSS Inc., Chicago, IL, USA). The normality of numerical data was assessed using Skewness and Kurtosis tests, as well as graphical evaluations with histograms and Q-Q plots. The Kolmogorov–Smirnov test was applied for normality assessment. Normally distributed continuous variables were analyzed using the Independent Samples *t*-test, while non-normally distributed variables were compared using the Mann–Whitney U test. Categorical data were analyzed using Pearson’s chi-square test.

The required sample size was determined using G*Power (version 3.1.9.7, Heinrich-Heine-Universität Düsseldorf, Düsseldorf, Germany). The power analysis was conducted to detect an effect size of 0.5 with a two-tailed test, a significance level of 0.05, and a statistical power of 80%. The results indicated that a minimum of 52 participants per group was required [18].

Sensitivity and specificity of VAI and DAI for distinguishing between groups were evaluated using receiver operating characteristic (ROC) curve analysis. The optimal cutoff values were determined based on the Youden index, which maximizes the sum of sensitivity and specificity. A *p*-value < 0.05 was considered statistically significant.

## 3. Results

The mean weight of women in the PCOS group was significantly higher than that of women in the control group (66.86 ± 15.94 vs. 62.11 ± 11.18, *p* = 0.037). The average BMI and WC values of PCOS patients were also significantly higher compared to those in the control group (*p* = 0.015 and *p* = 0.034, respectively). The presence of insulin resistance (IR+) was detected at a statistically significantly higher rate in PCOS patients (*p* < 0.001). Table 1 presents the demographic and anthropometric values of both groups.

The levels of serum LH (*p* = 0.013), E2 (*p* = 0.029), total testosterone (*p* < 0.001), FINS (*p* < 0.001), TG (*p* < 0.001), and HOMA-IR (*p* < 0.001) were significantly higher in patients with PCOS compared to the control group. The FSH value in the PCOS group was significantly lower than that of the control group (5.59 (0.54–49.35) vs. 6.78 (3.12–16.83), *p* < 0.001). Table 2 provides a detailed comparison of biochemical parameters between the PCOS and control groups.

**Figure 2:**
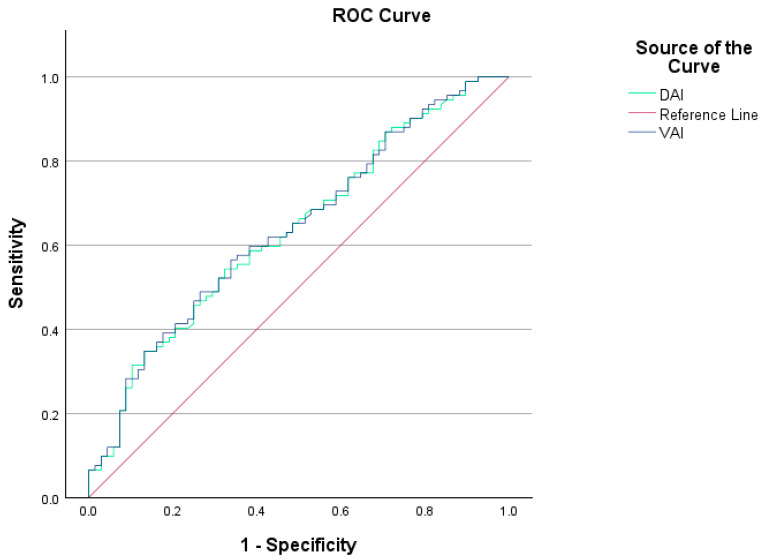
VAI and DAI ROC curves showing sensitivity and specificity values for individuals with and without PCOS.

The VAI (3.43 (1.03–18.15) vs. 2.16 (0.65–10.60), *p* = 0.003) and DAI (2.48 (0.72–10.05) vs. 1.54 (0.46–5.97), *p* = 0.003) values were significantly higher in the PCOS group compared to the control group. The ideal cutoff value for the VAI in predicting the diagnosis of PCOS was 3.10, with a sensitivity of 56% and a specificity of 66%. The AUC for the VAI was 0.639 (*p* = 0.001). The cutoff value for the DAI level was 0.22, with 54% sensitivity and 67% specificity, and an AUC of 0.635 (*p* = 0.002). It should be noted that the VAI and DAI indices were not statistically significant for diagnosing PCOS. Figure 2 presents the ROC curves for VAI and DAI in predicting a PCOS diagnosis.

**Figure 3:**
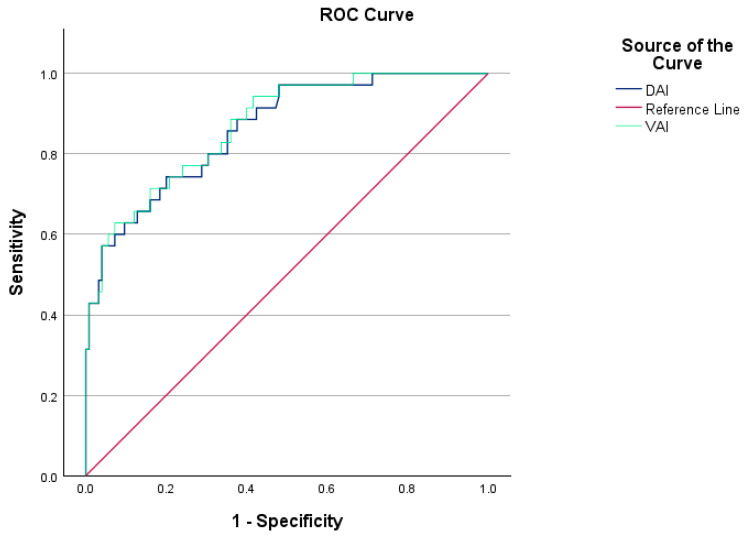
VAI and DAI ROC curves showing sensitivity and specificity values for PCOS patients with and without MetS.

Among PCOS patients diagnosed with MetS (MetS(+) PCOS), the VAI was significantly elevated compared to those without MetS (MetS(−) PCOS) (*p* < 0.001). The optimal cutoff value for VAI in predicting a MetS diagnosis in PCOS was determined to be 4.73, demonstrating a sensitivity of 62% and a specificity of 92%. The AUC for VAI in the prediction of MetS in PCOS patients was 0.870 (*p* < 0.001). Figure 3 presents the ROC curves for VAI and DAI in predicting MetS in PCOS patients.

Additionally, the DAI values were significantly higher in MetS(+) PCOS patients compared to MetS(−) PCOS patients (*p* < 0.001). The optimal cutoff value for DAI was determined to be 2.44, yielding a sensitivity of 74% and a specificity of 80%. The AUC for DAI in the prediction of MetS in PCOS patients was 0.862 (*p* < 0.001).

The VAI was significantly elevated in PCOS patients with IR (IR(+) PCOS) compared to those without IR (IR(−) PCOS) (*p* < 0.001). The optimal cutoff value for VAI in identifying IR in PCOS patients was found to be 2.56, with a sensitivity of 82% and a specificity of 56%. The AUC for VAI was determined to be 0.754 in the prediction of IR in PCOS patients (*p* < 0.001).

Likewise, the DAI value was statistically significantly higher in IR(+) PCOS patients compared to IR(−) PCOS patients (*p* < 0.001). The cutoff value for DAI in determining IR in PCOS patients was established at 1.59, demonstrating a sensitivity of 82% and a specificity of 55%, with an AUC of 0.749 (*p* < 0.001).

## 4. Discussion

This study investigated the efficacy of novel metabolic indices, specifically the VAI and DAI, in predicting insulin resistance (IR) and metabolic syndrome (MetS) among women with PCOS. Furthermore, this research aimed to determine whether VAI and DAI could serve as reliable biomarkers for the diagnosis of PCOS. The results indicate that VAI and DAI do not demonstrate sufficient specificity or sensitivity for diagnosing PCOS. Nonetheless, our findings reveal that VAI and DAI may serve as robust markers for screening IR and MetS in this population. Although prior studies have examined the association of VAI with metabolic risk factors, therapeutic outcomes, and IR in PCOS patients, this study is the first to assess the relationship between DAI and both the diagnosis of PCOS and its association with IR and MetS [19,20,21].

In our study, anthropometric measurements such as average weight (*p* = 0.037), BMI (*p* = 0.015), and WC (*p* = 0.034) were found to be significantly higher in women with PCOS compared to healthy controls, consistent with previous studies. IR is recognized as a fundamental component of the pathophysiology of PCOS, and its effects are exacerbated by obesity [22,23]. Accordingly, our findings demonstrated that IR was detected at a statistically significantly higher rate in PCOS patients compared to controls (*p* < 0.001). Similarly, MetS is a common metabolic complication among PCOS patients, as reflected in our study, where its prevalence was significantly higher in the PCOS group than in the control group (*p* < 0.001) [24].

Additionally, we observed that LH (*p* = 0.013), E2 (*p* = 0.029), total testosterone (*p* < 0.001), FINS (*p* < 0.001), TG (*p* < 0.001), and VAI (*p* = 0.003) were significantly elevated in the PCOS cohort. These findings align with other studies investigating biochemical parameter variations in PCOS [25,26]. Notably, this study is the first to assess DAI in PCOS, and it was found to be statistically significantly higher in the PCOS group compared to the control group (*p* = 0.003).

It is well established that visceral adipose tissue is strongly correlated with metabolic risk factors and IR. Conventional anthropometric measurements, such as BMI and WC, are insufficient for accurately reflecting visceral fat distribution. Women diagnosed with PCOS exhibit a higher prevalence of central obesity and an increased risk of metabolic disturbances [27,28]. Discrepancies in findings may arise from methodological biases inherent in the varying measurement techniques of anthropometric indices. Furthermore, the pattern of adipose tissue distribution may differ significantly among PCOS patients with identical BMI values. Consequently, there is an urgent need to develop more specific indices that integrate anthropometric assessments with biochemical markers to accurately characterize visceral adiposity and its associated cardiometabolic complications, including IR and MetS, in individuals with PCOS [29,30].

The evidence indicates that VAI is superior to BMI in predicting IR and MetS. Oh et al. initially proposed that VAI could effectively replace visceral computed tomography as a marker for visceral adiposity, demonstrating its predictive capacity for IR in young women with PCOS [31]. Visceral obesity affects 40–85% of women with PCOS, who also exhibit components of MetS, including IR, dyslipidemia, and hypertension. The VAI has emerged as a significant biomarker for the early detection of metabolic disorders in women with PCOS. However, a study conducted by Uysal et al. (2024) found no significant relationship between VAI values and IR(+) PCOS patients. Nonetheless, numerous studies, including ours, demonstrate that VAI is a strong indicator for identifying IR(+) and MetS(+) PCOS patients [21].

The optimal cutoff value for VAI in predicting a MetS diagnosis in PCOS was determined to be 4.73, with an AUC of 0.870 in our study. Additionally, VAI was identified as a successful marker for predicting IR(+) PCOS patients, with an optimal cutoff of 2.56 and an AUC of 0.754. However, VAI is not a purely biochemical parameter; its geographic and racial variations due to the parameters used in its calculation, such as WC and BMI, may contribute to variability in study outcomes [32].

DAI has been evaluated in a limited number of studies as a new marker reflecting adipocyte dysfunction. Reyes-Barrera et al. emphasized that DAI serves as a clinical surrogate for evaluating adipose tissue functionality and cardiometabolic health, reporting that DAI demonstrated good diagnostic values for detecting increased pericardial fat volume [15]. Mantovani et al. found that in a population including both men and women, a DAI ≥ 1.065 was independently associated with diabetes, non-alcoholic fatty liver disease, subclinical atherosclerosis, and hypertension. They also noted that DAI is associated with early cardiometabolic abnormalities independent of adiposity and other risk factors [33].

Although the lipid profile in PCOS patients was not drastically different from that in controls, it is crucial to consider that VAI and DAI incorporate additional metabolic parameters beyond lipid levels. The significant differences in waist circumference and BMI between groups suggest increased visceral adiposity in PCOS patients, which is a key driver of metabolic dysfunction. Furthermore, insulin resistance—a hallmark of PCOS—can lead to alterations in lipid metabolism and adipocyte function, even in the absence of overt dyslipidemia. Chronic low-grade inflammation and hormonal imbalances, particularly hyperinsulinemia and androgen excess, further contribute to adipose tissue dysfunction. These metabolic disturbances collectively explain why VAI and DAI remain elevated in PCOS patients despite a relatively similar lipid profile. This finding underscores the need for integrated metabolic indices that assess adipose dysfunction beyond conventional lipid measurements [9,25].

Given that DAI is a newly introduced clinical marker, previous studies have primarily focused on mixed-gender populations and have been conducted in Latin American and Hispanic communities [15,34]. In one study, 172,282 women were included, and DAI showed an AUC of 0.819 for MetS [35]. Jalali et al. claimed that DAI was independently associated with IR and β-cell dysfunction in patients at high risk for T2D. However, in their study, 71.6% of the patients were women, and the cutoff values were not specified separately for men and women [36].

Our study is the first to use DAI to predict MetS and IR positivity in PCOS patients. The optimal cutoff value for DAI was determined to be 2.44, with an AUC of 0.862 in predicting MetS in PCOS patients. The cutoff value for DAI in determining IR in PCOS patients was established at 1.59, with an AUC of 0.749 in our study.

This study has several limitations. First, the cross-sectional design prevents causal inferences regarding the relationships between VAI, DAI, and metabolic disorders in PCOS. Second, this study was conducted at a single center, which may limit its generalizability to broader populations. Third, important confounding factors such as physical activity, dietary intake, and socioeconomic status were not assessed, which could have influenced the findings. Finally, although our study provides valuable insights into the role of VAI and DAI in PCOS, further large-scale, multicenter, prospective studies are necessary to validate these findings and determine the optimal cutoff values for different populations.

## 5. Conclusions

In conclusion, VAI and DAI stand out as promising new metabolic markers for predicting IR and MetS in women with PCOS. Their use in clinical practice may enhance early detection of cardiometabolic risks, offering a more specific assessment compared to traditional anthropometric measures. However, especially for DAI, further prospective, multicenter studies in different populations are needed to support the use of these markers as routine screening tools for patients with PCOS.

## Figures and Tables

**Table 1:** The demographic and anthropometric values of PCOS group and control group.

	PCOS (100%/*n* = 92)	Control (100%/*n* = 68)	*p* Value ^a,b^
**Age (years)** **^Ω^**	22 (18–40)	29 (20–48)	**<0.001**
**Height (cm)** **^Ω^**	163 (120–175)	163 (149–179)	0.654
**Weight (kg)** **^Ω^**	62.5 (43–115)	60 (39–87)	**0.037**
**BMI (** **kg/m^2^) ^Ω^**	23.74 (17.3–48.61)	22.8 (15.17–33.2)	**0.015**
Underweight (<18.5)	6.5% (6)	8.8% (6)	0.087
Normal Weight (18.5–24.9)	45.7% (42)	58.8% (40)	
Overweight (25–29.9)	23.9% (22)	25.0% (17)	
Obese (30–39.9)	22.8% (21)	7.4% (50)	
Morbidly Obese (>40)	1.1% (1)	0.0% (0)	
**WC (cm)** **^Ω^**	86 (60–130)	78 (57–114)	**0.034**
**Hip circumference (cm)** **^Ω^**	103.5 (80–138)	100.5 (86–127)	0.085
**WHR** **^Ω^**	0.79 (0.66–0.94)	0.77 (0.65–0.94)	0.053
Low Risk (<0.80)	51.1% (47)	58.8% (40)	0.364
Medium Risk (0.80–0.85)	21.7% (20)	23.5% (16)	
High Risk (>0.85)	27.2% (25)	17.6% (12)	
**IR**			
(+)	46.7% (42)	20.6% (14)	**<0.001**
(−)	53.3% (50)	79.4% (54)	
**MetS** **^Ω^**			
(+)	27.2% (25)	14.7% (10)	0.059
(−)	72.8% (67)	85.3% (58)	

^a^ Independent Samples *t*-test, ^b^ Mann–Whitney U Test, ^Ω^ Median (lower–upper). BMI, body mass index; WC, waist circumference; IR, insulin resistance; MetS, metabolic syndrome; WHR, waist-to-hip ratio.

**Table 2:** Evaluation of biochemical parameters of individuals in PCOS and control groups.

	PCOS (*n* = 92/100%)	Control (*n* = 68/100%)	*p* Value ^a,b^
**FSH (mIU/mL)**	5.59 (0.54–49.35)	6.78 (3.12–16.83)	**<0.001**
**LH (mIU/mL)**	7.77 (0.43–57)	6.15 (2.76–16.13)	**0.013**
**E2 (pg/mL)**	38.39 (5–341)	50.53 (5–292.4)	**0.029**
**Total Testosterone (ng/dL)**	0.34 (0.05–0.89)	0.25 (0.03–0.65)	**<0.001**
**Prolactin (mIU/L)**	19.19 (0.65–68.38)	16.95 (8.22–42.76)	0.106
**TSH (mIU/L)**	1.87 (0.32–11.19)	1.91 (0.02–7.02)	0.918
**HbA1C (mmol/mol)**	5.05 (4.5–6)	5.10 (4.5–8)	0.499
**FINS (mIU/mL)**	11.08 (0.44–73.45)	7.72 (1.72–51.33)	**<0.001**
**TC (mg/dL)**	161.15 (0.25–284.4)	160.25 (105–237.2)	0.875
**LDL-C (mg/dL)**	85.05 (7.46–163)	89.18 (39.38–151.06)	0.865
**HDL-C (mg/dL)**	51.45 (26.9–97.8)	53.60 (28.6–90.4)	0.352
**TG (Mmol/L)**	91.40 (39.7–329.2)	70.65 (30.5–224.6)	**<0.001**
**FPG (mmol/L)**	90.60 (69.8–123)	91.15 (56.8–129.4)	0.556
**SBP (mmHg)**	109.00 (80–138)	106.00 (80–135)	0.542
**DBP (mmHg)**	69.00 (50–90)	67.50 (40–87)	0.918
**HOMA-IR (unit)**	2.45 (0.09–15.23)	1.46 (0.34–10.77)	**<0.001**
**VAI (unit)**	3.43 (1.03–18.15)	2.16 (0.65–10.60)	**0.003**
**DAI (unit)**	2.48 (0.72–10.05)	1.54 (0.46–5.97)	**0.003**

^a^ Independent Samples *t*-test, ^b^ Mann–Whitney U Test. FSH, follicle-stimulating hormone; FINS, fasting insulin; LH, luteinizing hormone; E2, estradiol; TSH, thyroid-stimulating hormone; FPG, fasting plasma glucose; HbA1c, hemoglobin A1C; TC, total cholesterol; HOMA-IR, homeostatic model assessment of insulin resistance; HDL-C, high-density lipoprotein cholesterol; TG, triglyceride; LDL-C, low-density lipoprotein cholesterol; SBP, systolic blood pressure; DBP, diastolic blood pressure; VAI, visceral adiposity index; DAI, dysfunctional adiposity index.

## Data Availability

The data that support the findings of this study are available from the corresponding author upon reasonable request.
